# From Ebola to COVID-19: emergency preparedness and response plans and actions in Lagos, Nigeria

**DOI:** 10.1186/s12992-021-00728-x

**Published:** 2021-07-09

**Authors:** Akin Abayomi, Mobolanle R. Balogun, Munir Bankole, Aduragbemi Banke-Thomas, Bamidele Mutiu, John Olawepo, Morakinyo Senjobi, Oluwakemi Odukoya, Lanre Aladetuyi, Chioma Ejekam, Akinsanya Folarin, Madonna Emmanuel, Funke Amodu, Adesoji Ologun, Abosede Olusanya, Moses Bakare, Abiodun Alabi, Ismail Abdus-Salam, Eniola Erinosho, Abimbola Bowale, Sunday Omilabu, Babatunde Saka, Akin Osibogun, Ololade Wright, Jide Idris, Folasade Ogunsola

**Affiliations:** 1 Lagos State Ministry of Health/Lagos Incident Management Command System, Lagos, Nigeria; 2 Lagos State Biosafety and Biosecurity Governing Council, Lagos, Nigeria; 3 College of Medicine University of Lagos, Idi-Araba, Lagos, Nigeria; 4 LSE Health, London School of Economics and Political Science, London, UK; 5 School of Public Health, University of Nevada, Las Vegas, USA; 6 Bloom Public Health, Lagos, Nigeria; 7 Global Emerging Pathogens Treatment Consortium, Lagos, Nigeria

**Keywords:** COVID-19, Disease outbreak, Emergency preparedness, Epidemic response, Nigeria

## Abstract

**Background:**

Lagos state is the industrial nerve centre of Nigeria and was the epicentre of the 2014 Ebola outbreak in Nigeria as it is now for the current Coronavirus Disease (COVID-19) outbreak. This paper describes how the lessons learned from the Ebola outbreak in 2014 informed the emergency preparedness of the State ahead of the COVID-19 outbreak and guided response.

**Discussion:**

Following the Ebola outbreak in 2014, the Lagos State government provided governance by developing a policy on emergency preparedness and biosecurity and provided oversight and coordination of emergency preparedness strategies. Capacities for emergency response were strengthened by training key staff, developing a robust surveillance system, and setting up a Biosafety Level 3 laboratory and biobank. Resource provision, in terms of finances and trained personnel for emergencies was prioritized by the government. With the onset of COVID-19, Lagos state was able to respond promptly to the outbreak using the centralized Incident Command Structure and the key activities of the Emergency Operations Centre. Contributory to effective response were partnerships with the private sectors, community engagement and political commitment.

**Conclusion:**

Using the lessons learned from the 2014 Ebola outbreak, Lagos State had gradually prepared its healthcare system for a pandemic such as COVID-19. The State needs to continue to expand its preparedness to be more resilient and future proof to respond to disease outbreaks. Looking beyond intra-state gains, lessons and identified best practices from the past and present should be shared with other states and countries.

## Background

Since the beginning of the twentieth century, there have been some notable pandemics including the 1918 ‘Spanish’ Flu caused by an influenza A(H1N1) virus, 1957 influenza A (H2N2) virus, 1968 influenza A (H3N2) virus, 2002 Severe Acute Respiratory Syndrome (SARS) caused by SARS coronavirus, and the 2009 Swine Flu caused by an influenza A(H1N1) virus [1, 2]. While not declared a pandemic in itself, the West African Ebola Virus Disease (EVD) epidemic between 2013 and 2016 was an outbreak with huge regional significance, claiming an estimated 11,300 lives in total [3]. In December 2019, the Coronavirus disease (COVID-19) caused by infection with the Severe Acute Respiratory Syndrome Coronavirus 2 (SARS-CoV-2) emerged in Wuhan city, China, and since then there has been a global spread of the disease to pandemic proportions [4]. The first documented case of COVID-19 in Africa was reported on 14th February, 2020 in Egypt, followed by Algeria on 25th February, 2020, and Nigeria on 27th February, 2020 [5, 6]. This first case in Nigeria was an Italian citizen who flew into the commercial city of Lagos from Milan. By 11th March 2020, the World Health Organization (WHO) had declared the COVID-19 outbreak a pandemic [7]. Now, it is well established that the disease is transmitted via respiratory droplets and close prolonged personal contact with infected persons or surfaces with the virus [8]. As of 5th July 2021, over 183 million cases had been confirmed, including almost four million deaths globally. The African continent accounts for 2.3% of confirmed COVID-19 cases and about 2.5% of deaths reported worldwide [9].

Lagos state, located in the southwestern region of Nigeria and the industrial nerve centre of the country, has been the epicentre of the COVID-19 pandemic in the country, similarly as it was for the EVD outbreak in 2014 [10, 11]. With a population of about 21 million, it is classed as a megacity and has the highest population density in Africa [12, 13]. Lagos was the entry point of the index case of EVD and thus there were already concerns about the entry of COVID-19 into Lagos before it was declared a pandemic and the State’s capacity for response [14]. Measures to manage the outbreak of a novel disease such as COVID-19 are reliant on a State’s existing operational readiness and capacities to prevent, detect and respond to public health emergencies [15].

As has already been well established, many of the emerging epidemic and pandemic threats that confront humanity have a zoonotic origin [16]. The timing of these threats might be difficult to predict but their emergence at some point in time is guaranteed. As such, any plans to mitigate such threats need to be robust and ‘ready-to-go’. The WHO defines emergency preparedness as “the knowledge, capacities and organizational systems developed by governments, response and recovery organizations, communities and individuals to effectively anticipate, respond to, and recover from the impacts of likely, imminent, emerging, or current emergencies” [17]. This article describes the efforts of Lagos State in developing a preparedness and response plan based on lessons learnt from the 2014 EVD outbreak and the deployment of this plan ahead of the COVID-19 outbreak in the state. The article then critically analyses this plan and the corresponding response using the WHO’s Strategic Framework for Emergency Preparedness which highlights governance, capacities, and resources as the elements of emergency preparedness [16].

## Lagos State’s implementation approach for emergency preparedness

**Fig. 1:**
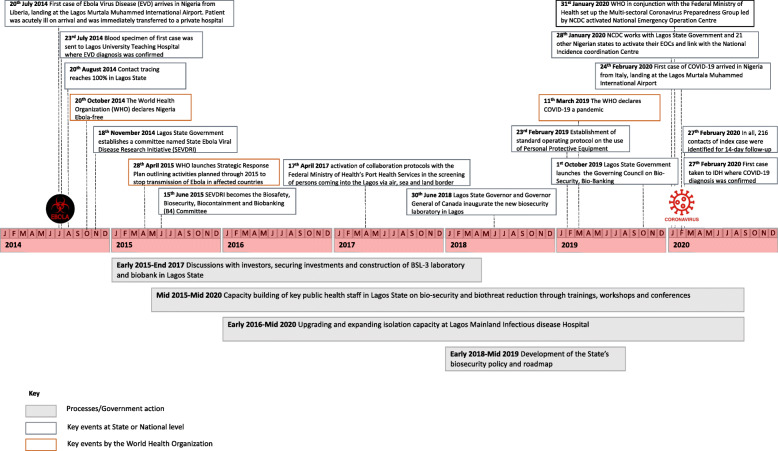
Timeline of Lagos State’s epidemic preparedness following the 2014 EVD outbreak

This section maps the key events that occurred between the emergence of EVD and that of COVID-19 along with implementation actions of the Lagos State Government during this period (Fig. 1). The section describes these actions in line with the three key elements of emergency preparedness, as recognized (governance, capacities, and resources) along with their sub-components [16].

### Governance

#### a. Policies and legislation that integrate emergency preparedness

Following the EVD outbreak in 2014, there was a clear need for Lagos state to develop a preparedness plan to be activated in the instance of any future outbreaks. A committee named State Ebola Viral Disease Research Initiative (SEVDRI) was established in November 2014. SEVDRI was mandated to conceptualise an emergency preparedness framework aimed at increasing the State’s resilience and capacity to respond to biological and other public health threats. The committee working as technical partner with Global Emerging Pathogen Treatment (GET) Consortium, an international collaboration developed during the EVD outbreak to ensure that sub-Saharan Africa was prepared for any public health emergency of international concern by providing strategic recommendations and establishing infrastructure and research capacity [18], concluded that in addition to robust public health and surveillance systems, there was a need for policies and guidelines specific to biobanking and biosecurity in Lagos State. On the one hand, biobanking refers to the collection of biological material and the associated data and information stored in an organized system for a population and is critical to gain better understanding of emerging and their pathogens [19, 20]. On the other hand, biosecurity is a strategic and integrated approach to analyse and manage relevant risks to human, animal and plant life and health and associated risks for the environment [21].

A rapid review of the extant laws, policies, and regulations in the state was therefore conducted. The review was interested in any law or policy concerning the domestication of elements by virtue of Nigeria being signatory or aligned with the International Health Regulations [IHR (2005)], Practices of Veterinary Services, the Biological Weapons Convention, the United Nations Security Council resolution 1540 and the Global Health Security Agenda. This task was approached with the mindset to propose and request amendments to current legislative acts relevant to biosecurity, if applicable, and to guide the development of a standalone emergency preparedness, biosecurity and biobanking bill. The review showed that at the time there were no current policies or legislation that could support emergency preparedness, biobanking or the threats that modern biotechnology pose to global health security such as the development, production, stockpiling, or use of biological weapons.

Subsequently, SEVDRI metamorphosed into Biosafety, Biobanking, Biosecurity and Biocontainment (B4) committee with an expanded and multi-pronged approach to: (a) work with the Lagos State legislature to identify gaps in legislation relating to emergency preparedness, biobanking and biosecurity in the state; (b) establish a framework and strategy for implementing biobanking and biosecurity in Lagos State with respect to preparedness and rapid response, referred to as the Lagos State Biosecurity and Biothreat reduction road map; and (c) establish the Lagos State Biosecurity and Biobanking Governance Council (LSBBGC), an entity mandated to constitute itself with diverse range of expertise whose sole responsibility is to assist the state to protect its biological space to ensure that it is healthy whilst also ensuring that knowledge that is derivable from it is sovereign and protected. This LSBBGC was set up to facilitate a scale-up of emergency preparedness against emerging infectious diseases [22]. The 12-member committee includes experts in bio-banking, biosecurity, molecular biology, public health, ethics, security, law, anthropology, environment, agriculture and representatives of the civil society [22].

**Fig. 2:**
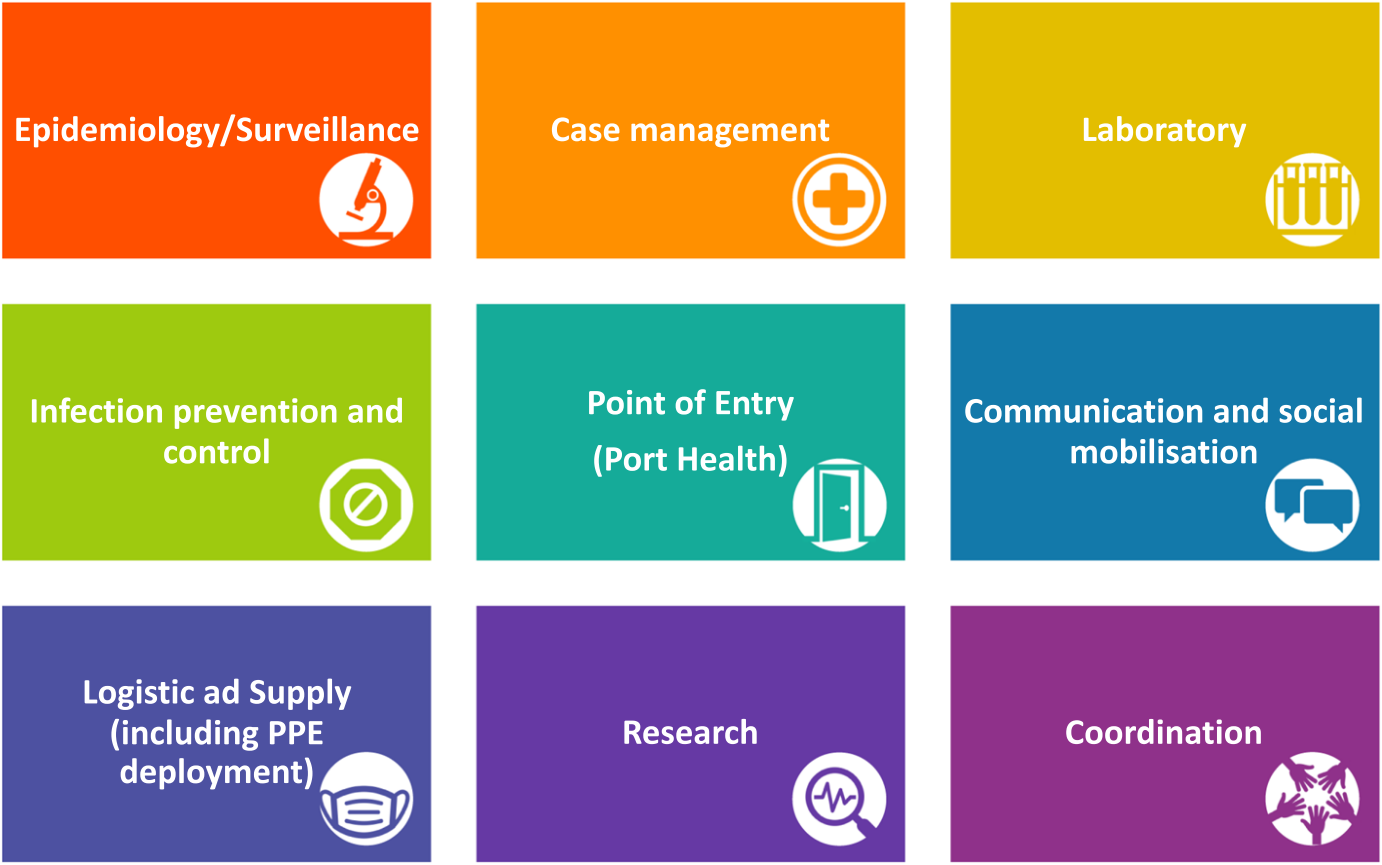
Units of Lagos State’s Emergency Operations Centre

Furthermore, the B4 committee drew up a draft policy on emergency preparedness, biobanking and biosecurity which went through several stakeholder engagements and was reviewed and validated by the LSBBGC and the relevant stakeholder ministries. This process was facilitated by the GET Consortium, an African-led multidisciplinary team of experts who are consultants to the Lagos State Government (LASG) on Biosecurity issues [18]. The policy built on existing State public health infrastructure such as the Lagos State Emergency Management Agency, Lagos State Ambulance Service, Lagos State Waste Management Authority, Lagos Mainland Infectious Disease Hospital (IDH), State Environmental Health Monitoring Unit and the Integrated Disease Surveillance and Response (IDSR) system at both the State and Local Government levels for routine surveillance and notification. Lagos State completed the five-year Biosecurity policy and roadmap in 2018 (Fig. 1). The revamped policy also leveraged experience garnered in tackling previous emergencies and epidemics [23]. For example, during the 2014 EVD outbreak, the state had set up its Emergency Operations Centre (EOC) which had six units focused on epidemiology and surveillance, communication and social mobilisation, case management and infection prevention control (IPC), laboratory services, point of entry (port health), and management and coordination [24]. These six units were subsequently expanded to nine units, making case management a separate unit from IPC and adding logistics and supply (including deployment of PPEs) as well as research (Fig. 2).

#### b. Plans for emergency preparedness, response and recovery

The EVD research committee that was set up during the EVD response was integrated into the B4 committee with the responsibility of overseeing the establishment of the emergency preparedness infrastructure, the Biosafety Level 3 (BSL-3) laboratory and the biobank facility, now referred to as the Lagos State Biobank (Fig. 1). The establishment of the biobank was supported by the LASG and the Government of Canada. As a unit, it has the only functional BSL-3 laboratory in Nigeria and it is the only energy mix hybrid biobank in West Africa, with its use of solar power as its primary source of energy deemed to be environmentally friendly [25, 26]. The biobank has a staff strength of 12 people including an infectious disease specialist (virologist), a director, a quality assurance manager, information technology (IT) staff, and a risk assessment officer. These staff make up the core of the Biobanking and Biosecurity Team (BBT), which is technically supported by the GET Consortium [18].

Advocacy continued following the 2014 EVD outbreak and significant investments were made in rapidly institutionalising emerging infection, prevention, and control procedures including the establishment of standard operating procedures on the use of Personal Protective Equipment (PPE), activation of collaboration protocols with the Federal Ministry of Health’s Port Health Services for screening persons arriving in Lagos via its land, sea, and air borders. The state’s Ministry of Health also designated the IDH as the referral facility for suspected or confirmed cases of any emerging infectious disease. In addition, the government directed all public health facilities to set up a dedicated isolation ward for suspected cases of Ebola and Lassa fever (the infectious disease of concern at the time), and to provide healthcare workers with a buffer stock of PPE [23].

#### c. Coordination mechanisms

The LSBBGC was tasked with providing oversight and coordination of the different strategies for biosecurity. This council has decision-making responsibilities for both technical and governance issues throughout the process of advancing the emergency preparedness and biosecurity program. The council went through a year-long orientation in 2018. This was seen as a critical activity and a key pillar to the long-term success of the Lagos State emergency preparedness and biosecurity framework.

The LASG via the Directorate of Epidemiology, Biosecurity and Global Health, inaugurated the Epidemic Preparedness and Response (EPR) committees and Rapid Response Teams (RRT) in each local government area (LGA) of the state. The EPR committee in each LGA was given the mandate to develop and oversee the implementation of emergency preparedness strategies, action plans and procedures [27]. This committee consisted of the LGA health team and the manager of the local government council. The LGA health team included the Medical Officer of Health, apex chief nursing office, apex community health officer, apex monitoring and evaluation officer and assistant, disease surveillance and notification officer (DSNO) and assistant, and the local immunisation officer.

The RRT is a technical and multi-disciplinary team that is readily available for quick mobilisation and deployment in cases of emergencies in line with guidelines of the Federal Ministry of Health [27]. In each LGA, members of the team included the LGA chairman, the head of the community development committee, one divisional police officer, the head of the neighbourhood security commission, the head of works and housing, and all members of the LGA health team.

### Capacities

#### a. Assessments of risks and capacities to determine priorities for emergency preparedness

Following the successful management of the 2014 EVD outbreak, LASG commenced the process of increasing its capacity to manage and contain future biosecurity threats. Between 2015 and 2020, LASG in collaboration with GET consortium, convened five biosecurity conferences across the sub-region of West Africa and trained its key staff in biosecurity and biothreat reduction (Fig. 1) [28].

The LSBBGC and the BBT also set out to ensure that the Lagos State biobank met international standards. This was achieved by benchmarking the Lagos State biobank with the biobank in the University of Stellenbosch, South Africa, using all the governance mechanisms already established by collaborating consortia on genomics and bioinformatics: H3Africa, B3Africa and GET Consortia [29, 30].

Additionally, a risk assessment plan was developed using a One Health approach [23, 31]. Supported by the WHO, the emergency preparedness team carried out routine risk assessment by checking for microbiological organisms in water bodies.

#### b. Surveillance, early warning and information management

In 2014, the Nigeria Centre for Disease Control (NCDC) and the Lagos State Ministry of Health (LSMOH) established an Incident Management Centre (IMC) in Lagos State. This IMC was the implementing arm of the national response to EVD and was renamed as the National EOC in the same year. The EOC took an incident management approach to identify and rapidly respond to threats of disease outbreaks [10]. It is a central point of assembly and coordination for response activities during public health events [17]. The EOC in Lagos State was the forerunner of the 20 EOCs in Nigeria as of year 2019 [23].

Additionally, a more robust surveillance system was developed where each LGA has DSNOs who meet every month to report on surveillance activities in their LGAs. These meetings, funded by the LASG and supported by the WHO, also provided an opportunity to discuss the challenges with the surveillance and reporting systems.

#### c. Access to diagnostic services during emergencies

The LASG pioneered laboratory biosecurity efforts in Nigeria by being the first state in the country to design and construct a BSL-3 laboratory. This lab has the capacity to run 1500 PCR tests daily and is building genomics and bioinformatics capacity [32].

### Resources

#### a. Financial resources for emergency preparedness and contingency funding for response

The state has adequately funded both the emergency preparedness activities and the biobanking agenda. This funding has supported several capacity building sessions, infrastructural upgrades, regular meetings, and a strong surveillance and reporting structure. Funding for preparedness efforts was also sourced from foreign governments and agencies. For example, the biobank which was delivered through a partnership between LASG and the Canadian government, through its Global Partnership Program (GPP), which contributed to the US$4.5 million project [33].

#### b. Dedicated, trained and equipped human resources for emergencies

Since the 2014 EVD outbreak, several LSMOH staff have attended workshops and certification trainings on emergency preparedness, biosafety, biosecurity, infection prevention and control, data governance, and sample governance. These trainings were conducted by GET Consortium, WHO, Public Health England, South African National Biodiversity Institute, Stellenbosch University, and Western Cape University.

## Outcome of Lagos’ emergency preparedness plan in the wake of COVID-19

### COVID-19 response plan

Lagos State articulated a COVID-19 Response Plan in June 2020 that identified and described the systems, activities, resources, and timelines required to combat the COVID-19 pandemic at the State and LGAs. The response plan builds on the existing capacities created by the responses to recent outbreaks in the State, taking into account lessons learnt from those previous outbreaks while not being oblivious to the peculiarity of the current threat.

**Fig. 3:**
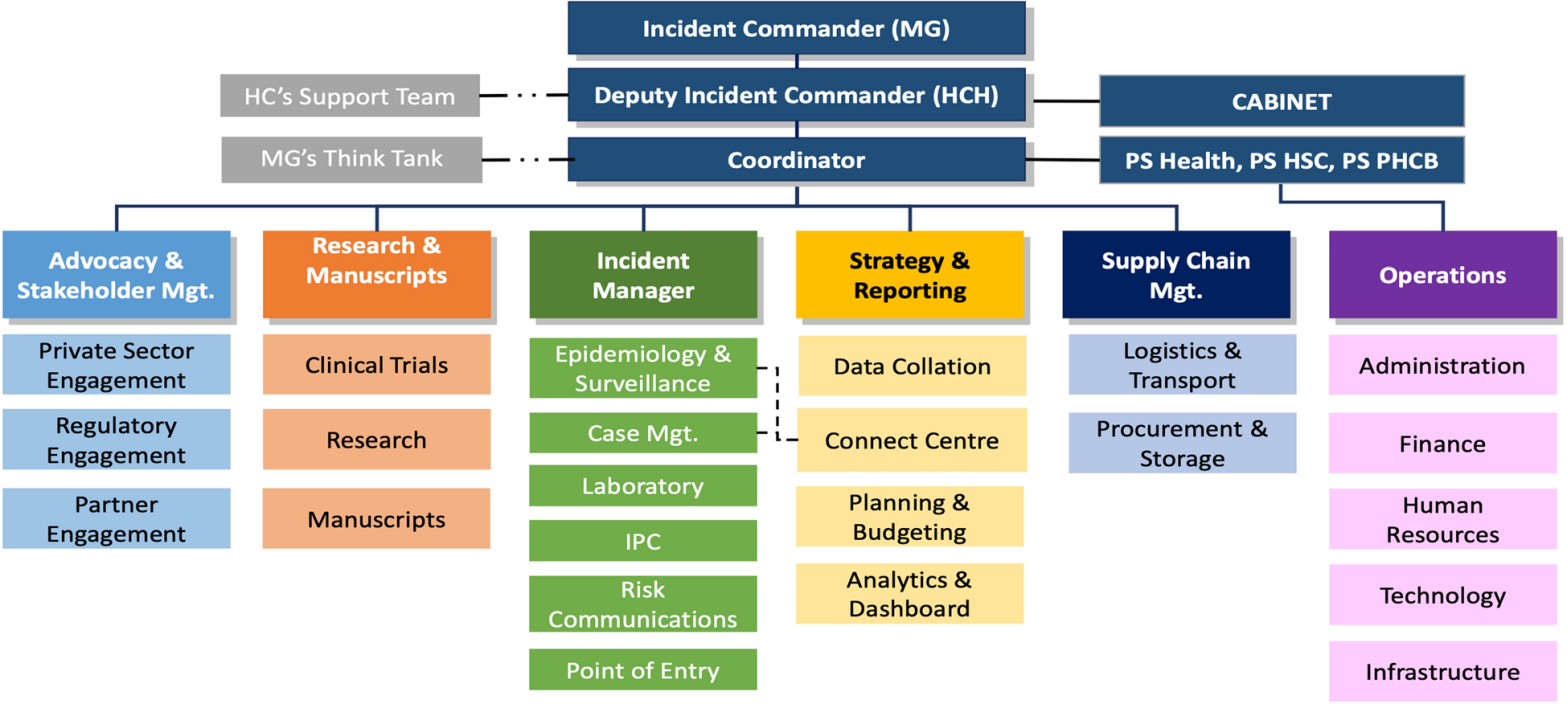
Structure of the Lagos State Incidence command system

The COVID-19 response plan is being implemented by the EOC. Four weeks before the index case, Lagos state set-up an Incident Command System (ICS), the body responsible for defining the strategic direction of the State’s response to the COVID-19 pandemic. The ICS is led by the Governor of Lagos State who serves as the Incident Commander, assisted by the state’s Commissioner for Health. The EOC is actively supported by local (NCDC) and international partners (WHO) who assist the operational pillars with their experience and capacity. The ICS is organized into six thematic areas: Advocacy and stakeholder management; Research and manuscripts; Incident Manager; Strategy and reporting; Supply chain management and Operations (Fig. 3) [21].

### Role of the think tank of the Lagos state incidence command system

As part of response to COVID-19, the Governor and Incident Commander created a COVID-19 Think Tank (Fig. 3). This was a human resource strategy involving a multidisciplinary team of subject matter experts who continuously interrogate the entire response direction and provide continuous guidance to the State on process improvement. The Think Tank facilitates timely decision making, dissemination of information to the government, the media, interest groups involved in the public policy process as well as the public. It is a dynamic team which includes experts in governance, research, health management information systems, donor engagement, technical assistance, infection prevention and control, psychosocial interventions, public relations, and communications. The guidance they provide are in broad areas of coordination and project management, strategy, clinical governance, research, communication, and logistics.

### Response activities

Mobilisation of resources and responses was made easier because of the ICS was already in place before the first case of COVID-19 in Lagos/Nigeria. For instance, the diagnosis of the first COVID-19 case in Lagos/Nigeria was made within six hours of arrival at IDH compared to three days to make the diagnosis of the first EVD case in 2014 [28]. This first case was a 44-year-old man who flew into Lagos 24th February 2020 on a Turkish Airline flight from Milan. The man stayed at a hotel near the airport the night he arrived and was driven to his workplace at Ewekoro, Ogun state, which shares a boundary with Lagos state, the day after. He felt ill after two days and reported at a clinic [34].

The EOC was activated immediately at the State and at LGA levels, to ensure the grassroots penetration of all activities being carried out in response to the threat. All the activities were in alignment with guidelines issued by the National Centre for Disease Control (NCDC). These measures along with outcomes achieved between February and May 2020 are presented below:

i. Point of entry: there were screening of passengers at the airports, seaports, and land borders using temperature checks and enquiry about COVID-19 symptoms; infection prevention and control training for point of entry staff of distribution of IEC materials to staff, stakeholders, and passengers.
ii. Risk communication: this included sensitization meetings with the private sector, religious organizations, stakeholders in the educational sector, informal sector, and government agencies & parastatals. In addition, several trainings and capacity building were carried out. The trainings were targeted at market men and women, youth organisations, social mobilization committees, ward health committees, head teachers and principals, community-based organisations, artisans, faith-based organisations and traditional birth attendants. Between February and May 2020, focus group discussions (FGDs) were held across the 376 wards in the state with ten representatives in each ward. The result of the FGDs was used to refine the content of the various risk communication messages that had been produced. Market and motor park sensitization events were held in 125 markets and 25 motor parks across 20 LGAs and 37 LCDAs. There were also several press appearances on major television stations in local languages.
iii. Surveillance and epidemiology: Four hundred DSNOs were trained on case definition, active surveillance, contact tracing, case investigation and other reporting tools. Also, contacts of confirmed cases were traced, identified, and monitored for the period of the longest known incubation period of the virus. Active case search for COVID-19 was done across the whole state in which samples were collected from community residents that fit the case definition for suspected cases and 24 LGA walk-in sample collection sites were set up across the state to scale-up testing for COVID-19. During the period, 22,000 passengers onboard a flight with a COVID-19 confirmed case and 432 returnees were followed up across the State. Also, 3,200 persons who are contacts of confirmed cases were traced, identified and monitored for the period of the longest known incubation period of the virus. In addition, 241 health workers were identified and investigated for exposure to COVID-19.
iv. IPC: Sixteen IPC SOPs and guidelines developed and disseminated to relevant stakeholders. Clinical and non-clinical personnel were trained on IPC protocols and tracked for health care workers infection. Also, public and private health facilities have been assessed across the state for COVID-19 preparedness. During the period, 2972 personnel were trained (694 non-clinical, 2278 clinical) on IPC protocols.
v. Logistics: A supply chain strategy was developed, which outlined the flow of commodity and information, warehousing and distribution arrangement, Human resource as well as steps for emergency procurement. Also, a data management plan was developed to inform decision making on the replenishment and procurement of critical and essential commodities for the COVID-19 response. Furthermore, the Logistics Management Information System, Combined Daily Stock Status Dashboard, Emergency Procurement Process and COVID-19 supply chain were developed.
vi. Laboratory services: The State enhanced its capacity to provide laboratory support for the diagnosis of COVID-19 by improving the testing capacity of its laboratories from 180 samples at the beginning of the outbreak to 2000 samples a day across six laboratories including Lagos State Biobank and 54gene laboratory three months later. In particular, the Lagos State Biobank, a BSL-3 Laboratory, which was activated for COVID-19 testing in April 2020 [32], became pivotal to COVID-19 diagnosis in the state being responsible for 60% PCR-testing for COVID-19 diagnosis. Additional human resources were recruited and trained to support the activities of the laboratories across the State, ensuring that they can provide 24-h services. Also, standard operating procedures and job aid were developed to standardise procedures across all laboratories in the State.
vii. Case management: The political support and leadership from the government resulted in a favourable climate for public–private partnership with some banks and private organizations, which led to the construction and equipping of isolation centres in a short amount of time as well as supply and distribution of personal protective equipment to health facilities [28]. In doing this, the state was able to increase the number of its isolation centres from one to six and from 115 beds at the IDH to a total of 590 beds.

Budget to achieve these measures were drawn quarterly to cater for the uncertainty associated with the ever-changing COVID-19 pandemic. For the second quarter (May–July 2020), a budget of N10.8 billion (US$28.7 million) was estimated to actualise this incident action plan. The major costs were allocated tor case management (N5.1 billion (US$13.3 million)), laboratory services (N1.6 billion (US$4.2 million)) and human resources (N1.5 billion (US$4.0 million)) [28].

## Appraising implementation of the state’s emergency preparedness and response

As of 17th May 2021, Lagos has reported 58,713 laboratory confirmed cases of COVID-19, 56,990 have been discharged and 439 patients have died [11]. Modelled estimates showed that without robust response measures, significantly large number of cases and deaths should have been expected in Lagos and Nigeria [35–37]. This would have had grave consequences on an already overwhelmed health system, as has been reported for many parts of Africa [38, 39]. Some of the response measures that appear to have worked in stemming this tide include strict lockdowns, social distancing, including bans on social and religious gatherings and restrictions of intra- and inter-state movements [40]. Many countries in sub-Saharan Africa were swift in deploying similar response measures with similar successes although testing capacities remain inadequate in the region [41].

The fact that the EOC was in place in Lagos State before the pandemic started helped to get an advance party started as the virus reached Lagos. For example, the management of the index case was immediately able to commence at the IDH after laboratory confirmation and contact tracing commenced immediately [42]. Similar capacity for early diagnosis, swift isolation of cases and prompt contact tracing were strong points of the 2014 EVD outbreak response that led to only eight mortalities in Nigeria [10, 24]. At the time, the WHO described Nigeria’s response as a “spectacular success story”, as the total number of cases remained 19, with seven deaths [43]. However, with COVID-19, the entirety of the EOC was not in place and many members were just only commissioned when the outbreak reached Lagos. Critically, community and religious leaders were only brought on board after the committee had been established. It is established that community and religious leaders are huge influencers during outbreaks and play a pivotal role in the whole-of-society pandemic readiness that the WHO recommends for national responses [44, 45].

Health worker preparedness for COVID-19 in Lagos focused on capacity building. However, since the onset of the outbreak, health workers have faced several challenges including burnout from overwhelming workload, with some advocating for inclusion of coping strategies for optimizing mental health to be included in training activities [46]. This is an area that could be improved to ensure that frontline health workers are able to best provide the care that patients need in such crisis.

By partnering with private sector, Lagos was able to minimize some of the cost that would have been incurred in responding to the outbreak. A key example of this was the coming together of private sector collaborators to form the Coalition Against COVID-19 (CACOVID) which is providing financing for immediate purchase of medical supplies and the creation of isolation centres for patient care. For instance, one of the private sector collaborators was a financial institution, which worked quickly to transform a stadium into a 110-bed isolation centre within five days in partnership with Lagos State [47]. This is a huge strength for robust response, as shown in China where strengthening of domestic linkages was deemed key in responding to the pandemic [38]. A systematic review on health security capacities of 182 countries clearly showed that countries vary widely in terms of their capacity to prevent, detect and respond to outbreaks [15]. While governments in high income countries have the financial mettle to fund such capital-intensive structures, LMIC governments are not in such strong positions. Therefore, private sector support is indeed invaluable. Similar to Nigeria, Uganda, South Africa and Ghana were able to manufacture low-cost ventilators through public private partnerships. Senegal’s cheap and rapid COVID-19 diagnostic test was also enabled by the private sector [41].

Kapata and colleagues iterated that African countries are on heightened alert to detect and isolate cases of COVID-19 as substantial progress has been made since the 2014 EVD outbreak with lessons learnt from previous and ongoing outbreaks in addition to significant investments into surveillance and preparedness [6]. Lagos State recognised the risk and initiated planning for COVID-19. While some of its preparedness were framed based on lessons and experiences of the 2014 EVD outbreak, many were still being learnt on-the-go during the ongoing pandemic. The establishment of Incident Command Centre for outbreak preparedness and response activities has enabled the state to gather intelligence reports daily as well as identify impending public health threats and ensure that outbreak responses are well coordinated and controlled [48]. For the 2014 EVD outbreak, the success recorded in its control were attributed to the incident management approach with significant support provided by the private sector and international community [24]. However, with all countries including donor countries facing significant challenges of their own with COVID-19, the international community was understandably not particularly a huge contributor in the middle of the crisis. Indeed, private sector and personal philanthropic donations became important sources of funding for health facilities during the pandemic [49].

A key part of the strategy that has been added now is research. This has allowed for more targeted understanding of the pandemic and expansion of innovation in responding to it. For example, a survey of the symptoms and signs of the first 2,184 PCR-confirmed COVID-19 patients [50], provided evidence that shaped training of health workers on identification of COVID-19 cases.

## Conclusion

As with the 2014 EVD outbreak, many anticipated as “urban apocalypse” with COVID-19 infiltrating the Lagos megacity [43]. This did not happen then and is yet to happen with the ongoing pandemic. Indeed, preparedness is a continuous process in which action, funding, partnerships, and political commitment at all levels must be sustained. It relies on all stakeholders working together effectively to plan, invest in, and implement priority actions. Like other geographical entities that have had devastating outbreaks, Lagos State harnessed lessons learned from the 2014 EVD outbreak, in what has been referred to by some authors as the ‘Ebola legacy’ [51]. More importantly, many systems were already in place to tackle any outbreaks that occurred post-2014. The state’s response has also benefitted from strong leadership, robust collaborations with national and international partners, new partnerships with private sector and generous donations of altruistic individuals. However, the state needs to continue to expand its preparedness to be more resilient and future proof to respond to outbreaks like COVID-19, as it is clear that this is indeed the “new normal” for health systems. Looking beyond intra-state gains, lessons and identified best practices from the past and present should be shared with other states and countries.

## Data Availability

Not applicable.

## Abbreviations

EVD: Ebola Virus Disease
LASG: Lagos State Government
LSMOH: Lagos State Ministry of Health
LSBGC: Lagos State Biosecurity and Biobanking Governance Council
GET: Global Emerging Pathogens Treatment
BSL: Biosafety laboratory
BBT: Biobanking and Biosecurity Team
EPR: Epidemic Preparedness and Response
RRT: Rapid Response Team
LGA: Local Government Area
DSNO: Disease Surveillance and Notification Officer
IMC: Incident Management Centre
EOC: Emergency Operations Centre
ICS: Incident Command System
